# De Novo Reconstruction of Transcriptome Identified Long Non-Coding RNA Regulator of Aging-Related Brown Adipose Tissue Whitening in Rabbits

**DOI:** 10.3390/biology10111176

**Published:** 2021-11-13

**Authors:** Kun Du, Xue Bai, Li Yang, Yu Shi, Li Chen, Haoding Wang, Mingchen Cai, Jie Wang, Shiyi Chen, Xianbo Jia, Songjia Lai

**Affiliations:** 1 Farm Animal Genetic Resources Exploration and Innovation Key Laboratory of Sichuan Province, Sichuan Agricultural University, 211# Huimin Road, Chengdu 611130, China; dukun1672@163.com (K.D.); baixue333work@163.com (X.B.); ylyang1226@163.com (L.Y.); 18227551690@163.com (Y.S.); chenl2020302120@163.com (L.C.); W2458174617@163.com (H.W.); 20190027@cqwu.edu.cn (M.C.); wjie68@sicau.edu.cn (J.W.); chensy@sicau.edu.cn (S.C.); jaxb369@sicau.edu.cn (X.J.); 2 College of Landscape Architecture and Life Science/Institute of Special Plants, Chongqing University of Arts and Sciences, Chongqing 402160, China

**Keywords:** BATs, rabbits, ssRNA-seq, homeothermic ability, whitening

## Abstract

**Simple Summary:**

Brown adipose tissues (BATs) undergo the conversion to white adipose tissues (WATs) with age. Long non-coding RNAs (lncRNAs) were widely involved in adipose biology. Rabbit is an ideal model for studying the dynamics of the transformation from BATs to WATs. However, our knowledge of lncRNAs that mediate the transformation remains unknown in rabbits. By histological analysis and sequencing, we found rabbit interscapular adipose tissues (iATs) from BATs to WATs within two years and identified a total of 631 differentially expressed lncRNAs (DELs) during the transformation process. Several signal pathways were involved in the transformation from BAT to WAT. A novel lncRNA that was highly expressed in iATs of aged rabbits was validated to impair brown adipocyte differentiation in vitro. Our study provided a comprehensive catalog of lncRNAs involved in the transformation from BATs to WATs in rabbits, facilitating a better understanding of adipose biology.

**Abstract:**

Brown adipose tissues (BATs) convert to a “white-like” phenotype with age, which is also known as “aging-related BAT whitening (ARBW)”. Emerging evidence suggested that long non-coding RNAs (lncRNAs) were widely involved in adipose biology. Rabbit is an ideal model for studying the dynamics of ARBW. In this study, we performed histological analysis and strand-specific RNA-sequencing (ssRNA-seq) of rabbit interscapular adipose tissues (iATs). Our data indicated that the rabbit iATs underwent the ARBW from 0 days to 2 years and a total of 2281 novel lncRNAs were identified in the iATs. The classical rabbit BATs showed low lncRNA transcriptional complexity compared to white adipose tissues (WATs). A total of 631 differentially expressed lncRNAs (DELs) were identified in four stages. The signal pathways of purine metabolism, Wnt signaling pathway, peroxisome proliferator-activated receptor (PPAR) signaling pathway, cyclic guanosine monophosphate (cGMP)/cGMP-dependent protein kinase (cGMP-PKG) signaling pathway and lipid and atherosclerosis were significantly enriched by the DELs with unique expression patterns. A novel lncRNA that was highly expressed in the iATs of aged rabbits was validated to impair brown adipocyte differentiation in vitro. Our study provided a comprehensive catalog of lncRNAs involved in ARBW in rabbits, which facilitates a better understanding of adipose biology.

## 1. Introduction

The mammals contain white adipose tissues (WATs) that function as energy depositories and brown adipose tissues (BATs) that function in energy expenditure [1]. BAT is densely packed with mitochondria that express high levels of uncoupling protein 1 (UCP1), which allows proton leakage to uncouple respiration from ATP synthesis, playing an important role in thermogenesis for temperature homeostasis [1,2]. The BAT content is negatively correlated with the total fat deposition of the body [3,4]. In humans, BAT activity has beneficial metabolic effects on obesity, insulin resistance and atherosclerosis [5]. Although BAT can be found in most new-born animals, there is a difference in BAT development among different species [6,7,8]. The hibernating animal and some rodents persist in their BATs into adult life [9,10]. In most mammals such as ruminants, rabbits and humans, BATs undergo a poorly elucidated conversion to a “white-like” phenotype with age, which is known as “aging-related BAT whitening (ARBW)” [11]. However, the regulatory mechanisms underlying ARBW remain unknown.

Long non-coding RNAs (lncRNAs) are a type of RNA molecules that comprise >200 nucleotide residues and have no potential to code proteins [12]. Based on association with annotated protein-coding genes, lncRNAs can be classified into sense-overlapping lncRNA (SO lncRNA), antisense lncRNAs (AS lncRNAs), intronic lncRNAs and long intergenic non-coding RNAs (lincRNAs) [13]. LncRNAs play important roles in many different biological processes, such as tissue development, immune process and cancer formation [14,15,16]. On the other hand, several lncRNAs such as lnc-BATE1 [2], lnc-BATE10 [17], Blnc1 [18] and lnc-dPrdm16 have been recognized as important regulators for developing BATs in mice or humans [19]. Our previous studies identified that lncRNAs were widely involved in visceral WAT (vWAT) development and white adipocyte differentiation [20,21].

Rabbit (*Oryctolagus cuniculus*) is an economically important domestic animal due to its high-quality meat, fur and hair [22,23]. Rabbits have low-fat deposition than other mammals, such as swine, cattle and sheep [24]. The BAT content and activity may account for the natural low-fat deposition of rabbits. Thus, rabbit, as an animal model, can be used in studying the dynamics of ARBW. The BAT development is important for the temperature homeostasis of newborn rabbits, especially for those were born in the cold ambient temperature [10]. Investigation of the molecular mechanisms underlying the process of ARBW could contribute to improving the welfare and survival rate of newborn rabbits. However, our knowledge of lncRNAs that mediate ARBW remains largely unknown in rabbits.

Interscapular adipose tissue (iAT) is the major BAT depot of rabbits [10]. In this study, we carried out a histological analysis to determine the process of ARBW of iATs in rabbits. We performed strand-specific RNA sequencing (ssRNA-seq) to identify lncRNAs involved in the process. Our data revealed the development of ARBW in rabbits and a total of 2281 novel lncRNAs were identified in our samples. Furthermore, classical rabbit BAT was a low lncRNA transcriptional complexity tissue. We identified lncRNAs that were significantly correlated with their flanking or reference genes. Clustering analysis of differentially expressed lncRNAs (DELs) revealed that lncRNAs with different expression patterns in stages played different roles in a cis-regulating way during ARBW. One DEL, MSTRG.2316.1, was validated to impair brown adipocyte differentiation in vitro. Our work is the first report of the dynamics of lncRNA regulatory mechanisms underlying the ARBW in rabbits and facilitates a better understanding of adipose biology.

## 2. Materials and Methods

### 2.1. Ethics Approval

All surgical procedures involving rabbits were performed according to the approved protocols of the Biological Studies Animal Care and Use Committee, Sichuan Province, China. Rabbits had free access to food and water under normal conditions and were humanely sacrificed as necessary to ameliorate suffering.

### 2.2. Tissue Sample Preparation, Histological Analysis and Immunohistochemistry (IHC)

In this study, the Tianfu Black rabbits (native species in Sichuan province of China) were raised at the breeding center of Sichuan Agricultural University, Ya’an, China. These rabbits were given *ad libitum* access to a standard diet and water as described previously [20]. To determine the lncRNA dynamics during BAT development in rabbits, a total of 12 samples were collected from iATs at four growth stages of 0 days (D0, infant stage), 15 days (D15, early whitening stage), 85 days (D85, puberty stage) and 2 years (Y2, aged stage) under sterile condition and 3 individuals were set at each stage. The samples for ssRNA-seq were snap-frozen in liquid nitrogen and stored at −80 °C until RNA extraction. The samples for histological assay were fixed using 4% paraformaldehyde at 4 °C overnight, embedded in paraffin and sliced. Haematoxylin and eosin (H&E) staining was carried out after deparaffination according to standard protocols as described in our previous study [25]. For IHC, the slices were incubated in the primary UCP1 antibody (1:100, Sangon Biotech, Shanghai, China) at 4 °C overnight and washed three times using phosphate buffer saline (PBS). Then, the slices were incubated in the secondary antibody (1:500, rabbit anti-mouse IgG, Sangon Biotech, Shanghai, China) for 45 min. All slices including in the assays of H&E staining and the IHC, were observed using an Olympus BX-50F light microscope (Olympus Optical, Tokyo, Japan).

### 2.3. SsRNA-seq and lncRNA Identification

The ssRNA-seq was done following our previous study [20]. Briefly, the total RNA of these samples was extracted using TRIzol reagent (Invitrogen, Hong Kong, China) and 1 μg RNA was used to construct a strand-specific library using the deoxyuridine triphosphate (dUTP) method. All purified libraries were sequenced on an Illumina NovaSeq 6000 platform. Finally, 150 bp oriented paired-end reads were generated. The quality of ssRNA-seq reads was checked using the Fastqc program (v0.11.8) [26]. Sequencing adapters and low-quality reads were removed using Cutadapt (v3.2) [27] software with parameters of ‘-a AGATCGGAAGAGCACACGTCTGAACTCCAGTCAC -A AGATCGGAAGAGCGTCGTGTAGGGAAAGAGTGT -e 0.1 -m 100 --cut 0 -O 13’. Clean reads were aligned to the rabbit reference genome (OryCun2.0, Ensembl release 101) using HISAT2 (v2.1.0) [28] with the strand-specific parameter of ‘--rf’ and the parameter of ‘--dta’ for the downstream transcriptome reconstruction. Reconstruction of the transcriptome was conducted using Stringtie (v2.1.1) [29]. All transcripts generated from all samples were merged using Stringtie with a parameter of ‘merge’ to generate a consensus transcriptome. The transcripts with the length size of <200 bp were discarded and only transcripts containing multiple exons and having a value of transcripts per kilobase of exon model per million mapped reads >0.1 (TPM > 0.1) at least in one sample were collected to downstream analyses. To identify novel transcripts, the consensus transcriptome was compared to the rabbit reference transcriptome (OryCun2.0, Ensembl release 101) using Gffcompare (v0.11.2) [30]. The transcripts were located in the intergenic regions or a single intron, overlapping on an opposite strand of annotated transcripts and inadequately overlapping with an annotated exon were retained. For the retained transcripts, CPC2 (v0.1) [31], CPAT (v2.0.0) [32] and CNCI (2.0) [33] were used to check their protein-coding capacity and PfamScan [34] was used to align them to the Pfam database to remove potential protein-coding transcripts (http://pfam.xfam.org/, accessed on 10 November 2021). Only the non-coding transcripts identified by CPC2, CPAT, CNCI and PfamScan were considered credible lncRNAs. Most of the rabbit lncRNAs deposited in current databases had not yet been functionally annotated and lncRNAs shared poor conservation across species, which raised the challenges in inferring the functions of lncRNAs [35]. Recent studies demonstrated that lncRNAs could regulate the flanking genes to play their roles by cis-regulation [36]. We performed lncRNA category-based cis-regulation prediction. For AS lncRNA, SO lncRNA and intronic lncRNA, the protein-coding genes located at the loci of the lncRNA were considered potential cis-regulated target genes. For lncRNA, the protein-coding genes located within 100 kb of lincRNA were considered potential cis-regulated target genes. The potential cis-regulated targets were used to predict lncRNA functions.

### 2.4. Transcriptomic Quantification and Differential Expression Analysis

To identify DELs during ARBW of the rabbit iAT, we quantified gene expression levels of lncRNAs. The stringtie ‘-eB’ and ‘-A’ were used to estimate raw read counts and TPM. The raw read counts were used as inputs to identify DELs in pairwise comparisons over the time courses of D0, D15, D85 and Y2 using DESeq2 [37]. The P values of hypothetical tests were adjusted using the method of false discovery rate (FDR). The lncRNAs with the thresholds of |log2(fold-change)| > 1.5 and FDR < 0.01 were considered DELs. Based on the TPM value, all DELs were clustered using R software (v4.4.1) and the clustering result was visualized using R package complexHeatmap [38]. Kyoto Encyclopedia of Genes and Genomes enrichment (KEGG) pathway analysis was conducted using R package clusterProfiler [39] and the enriched pathways that had a *p* value < 0.05 were considered significant.

### 2.5. Cell Culture and Plasmid-Mediated Overexpression

The brown preadipocytes (BPAs) were isolated from iATs of new-born Tianfu black rabbits using the collagenase I (Gibco, Carlsbad, CA, USA) method as described in our previous study [21]. The BPAs were placed into 12-well plates at a density of 3 × 10^5^ cells per plate in the complete medium (Dulbecco’s Modified Eagle Medium (DMEM) with high glucose, supplemented with 10% fetal bovine serum (FBS)) (Gibco, Carlsbad, CA, USA) and BPAs were placed in a humidified incubator at 37 °C and 5% CO_2_. Upon reaching approximately 80% confluence, the cells were induced using differentiation medium I (DMEM with high glucose, supplemented with 10% FBS, 500 μM 1-methy1-3-iosbutylxanthine [IBMX], 10 μg/mL insulin, 50 nM T3 and 5 μM dexamethasone) for 2 days. The cells were cultured in differentiation medium II (DMEM with high glucose, supplemented with 10% FBS, 500 μM IBMX, 2.5 μg/mL insulin, 50 nM T3 and 1 μM rosiglitazone) for 2 days. Finally, cells were cultured in differentiation medium III (DMEM with high glucose, supplemented with 10% FBS, 500 μM IBMX, 50 nM T3 and 1 μM rosiglitazone) for an additional 1 day. The cells were treated with 10 µM isoproterenol for 4 h before harvesting. The IBMX, insulin, T3, dexamethasone and rosiglitazone were purchased from Sigma-Aldrich (Shanghai, China). The accumulated lipid droplets were measured by Oil red O staining. The lipid-combined Oil red O dye was extracted by 2.5 mL isopropanol. The optical density (OD-510 nm) of the Oil red O elution was used to quantify the degree of lipid accumulation.

For lncRNA overexpression (OE), the vector of pcDNA3.1(+) loaded MSTRG.2316.1 and empty vector were purchased from Sangon Biotech, Shanghai, China. BPAs growing in complete medium at approximately 80% confluence were transfected with 1.6 μg/mL concentration of MSTRG.2316.1 vector or empty vector using Lipofectamine 3000 reagent (ThermoFisher, Carlsbad, CA, USA) according to the manufacturer’s instructions. After eight hours, the cells were induced to differentiate (set day 0). The cells were harvested to detect OE efficiency at 48 h.

### 2.6. Quantitative Real-Time PCR (qRT-PCR)

Total RNA was extracted using Trizol reagent according to the manufacturer’s instructions. The qRT-PCR primers used were designed using online Primer3 software (Table S1). Total RNA was reversed transcribed to complementary DNA (cDNA) using PrimeScripts RT Reagent Kit containing gDNA Eraser (TAKARA, Dalian, China). Then, the cDNA template used for qPCR was determined using the SYBR II master mix kit (TAKARA, Dalian, China). The qPCR was performed on a Bio-Rad CFX manager according to the manufacturer’s instructions. The amplification reaction was conducted under the following program: pre-denaturation at 95 °C for 10 s, followed by 40 cycles of denaturation at 95 °C for 5 s and annealing/extension at 59 °C for 20 s. The melting curve analysis was performed from 65 to 95 °C with an increment of 0.5 °C. All the qRT-PCR Ct-values were normalized to the Ct-value of the ACTB gene and group D0 using the 2^−ΔΔCt^ method.

### 2.7. Statistical Analysis

Statistical analyses, including t-test and one-way analysis of variance (ANOVA), were conducted on R software. The *p* value < 0.05 was considered significant.

## 3. Results

### 3.1. Histological Dynamics of ARBW in Rabbits

**Figure 1:**
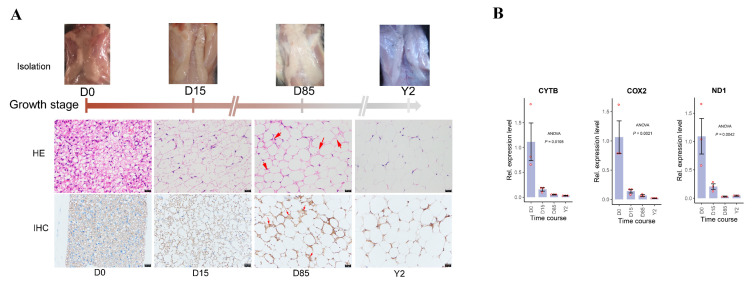
Histological characterization of ARBW in rabbits. (**A**) Isolation, H&E staining and immunohistochemistry (IHC) of rabbit iATs at four stages. The upper panel indicates the biopsy of the iATs, the middle panel indicates the H&E staining results of the iATs, the bottom panel indicates the IHC results of iATs. The scale bar: 20 μm. The red arrows in H&E and IHC results indicate the multilocular adipocytes in D85. (**B**) The expression levels of mitochondrial genes were detected at four growth stages during rabbit iAT whitening using qRT-PCR. The expression was normalized to the ACTB gene and D0. The data shows the means of three independent experiments. Two technical replicates were set for one individual experimental replicate. The “Rel.” represents “Relative”.

According to the histological analysis, the brownish color of iATs gradually faded during the development of rabbit iAT (Figure 1A upper panel). There was an increase in cell diameter from D0 to Y2 and obvious heterogeneity of adipose tissue was found at D15 and D85. The cells in D0 were composed of multiple small triglyceride droplets (multilocular adipocytes), while cells in Y2 were composed of a single large lipid droplet (unilocular adipocytes) (Figure 1A middle panel). The ratio of multilocular adipocytes to unilocular adipocytes gradually decreased from D0 to Y2 and the iATs in D85 contained a very low proportion of multilocular adipocytes (Figure 1A middle panel). The IHC assay showed that the expression levels of UCP1 protein gradually declined during the development of iATs (Figure 1A bottom panel). Furthermore, the results of the qRT-PCR assay showed that transcriptional copy numbers of the mitochondrial genes, including CYTB, COX2 and ND1, were dramatically decreased from D0 to D15 and then gradually decreased from D15 to Y2 in the tissue level (Figure 1B).

### 3.2. Identification and Characterization of lncRNAs in Rabbits iATs

**Figure 2:**
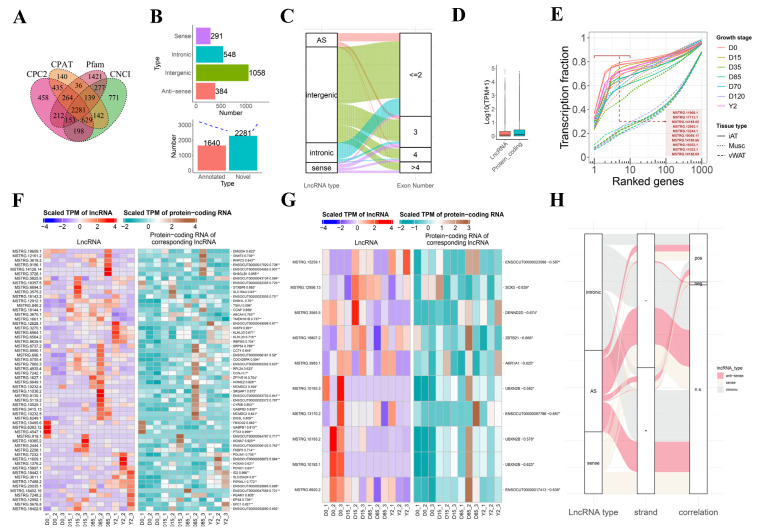
Genome-wide identification and characterization of lncRNAs during ARBW of rabbit iATs. (**A**) The Venn diagram of lncRNA transcripts from four types of software, including CPC2, CPAT, CNCI and Pfam. Only the lncRNAs identified by all the four types of software were used for downstream analyses. (**B**) Classification of novel lncRNAs and integration of novel lncRNAs and Esembl annotated lncRNAs expressed in the iATs. The upper panel indicates the classification of novel lncRNAs and the bottom panel shows total numbers of expressed Ensembl annotated lncRNAs and novel identified lncRNAs. (**C**) Alluvial diagram analysis of exon number of expressed lncRNAs shows that a higher proportion of SO lncRNA (purple stream) contains multiple exons. (**D**) Box plots of expression levels for lncRNA and protein-coding genes in rabbit iATs. (**E**) Comparison of transcriptional complexities among iATs, vWATs and skeletal muscle tissues. Every curve shows that the cumulated distribution of the fraction of total transcription contributed by lncRNAs when sorted from most to least expressed ones in each tissue. The different curve colors indicate different growth stages and different curve types indicate different tissue types of rabbits. iATs, interscapular adipose tissues. Musc, skeletal muscle tissues. vWAT, visceral white adipose tissues. The top 10 expressed lncRNAs of iAT in D0 are listed in the figure. (**F**,**G**) Heatmap analysis of AS lncRNAs and their anti-sense protein-coding genes. The left heatmap depicts the lncRNA expression pattern and the right heatmap depicts the anti-sense protein-coding gene expression patterns across samples. The Pearson’s correlation coefficients and *p* values of significance tests were marked following protein-coding gene symbols. The “*” represents *p* < 0.05 and “**” represents *p* < 0.01. (**H**) AS lncRNA that were negatively correlated with their corresponding protein-coding genes were prone to enrich in the negative strand of genome. The “pos” represents “a positive correlation with protein-coding genes” and “neg” represents “expression of lncRNAs negatively correlated with protein-coding genes” and n.s. represents “a negative correlation with protein-coding genes”. Most of the lncRNAs in “neg” came from AS lncRNAs that transcribed from negative strand (red stream).

To better define active lncRNAs during the ARBW of iATs, we reconstructed credible ted the transcriptome of iAT at the growth stages of D0, D15, D85 and Y2 in rabbits (n = 3 per stage). We performed paired-end ssRNA-seq for each tissue and obtained an average of 122.33 million clean reads from each sample, of which an average of 94.01% was properly mapped to the rabbit genome (Table S2). A total of 2281 novel lncRNAs were identified using different machine-learning models (Figure 2A). The novel lncRNAs were then classified into 4 categories, including lincRNAs (1058), SO lncRNA (291), intronic lncRNAs (548) and AS lncRNAs (384) (Figure 2B upper panel). By integrating the 1640 Esembl annotated lncRNAs (all were classified lincRNAs category), which were expressed in our samples, we obtained a total of 3921 lncRNAs expressed in the rabbit iATs (Figure 2B bottom panel). Analysis of rabbit iAT lncRNA structure found that most lincRNAs, intronic lncRNAs and AS lncRNAs contained one or two exons, especially for intronic lncRNAs, while more SO lncRNAs contained multiple exons (Figure 2C). When compared to protein-coding genes, lncRNAs were less expressed (Figure 2D), which were in line with our previous lncRNA study in rabbits [20,21] and other animals [2]. Analysis of lncRNA transcriptional complexity of the iATs, visceral white adipose tissues (vWATs) and skeletal muscle tissues revealed that the complexities of the of iATs and skeletal muscle tissues were lower than those of vWATs. The iATs in D0, which represented the classical BAT, were the least complex tissue (top 10 expressed lncRNAs, accounting for approximately 80% of total lncRNA expression). Our data showed that the top 10 expressed lncRNAs in BATs were novel lncRNAs, such as MSTRG.11968.1 (an intronic lncRNA at ZNF777), MSTRG.17113.1 (an AS lncRNA of MAPK8) and MSTRG.14188.65 (a lincRNA located at an unplaced genome scaffold) (Figure 2E). We characterized a catalog of credible novel lncRNAs expressed in the rabbit iATs and indicated that classical BATs were low lncRNA transcriptional complexity tissues.

We explored the expression correlation between lncRNAs and the protein-coding genes located at the corresponding lncRNA loci. For the AS lncRNAs, the expression patterns of 58 ones were significantly positively correlated with their anti-sense protein-coding genes across all samples (Pearson’s correlation coefficient, *p* < 0.05). For instance, the AS lncRNAs located at the loci of FTX3, ENSOCUT00000058972, ID2, KCNA7, ENSOCUT00000034065 were the top five lncRNA that had the highest correlation coefficients with their corresponding protein-coding genes (Figure 2F). On the other hand, 10 AS lncRNAs were significantly negatively correlated with their anti-sense protein-coding genes, such as the AS lncRNAs located at the loci of Ubiquitin regulatory X (UBX) domain protein 2B (UBXN2B), sex determining region (SRY) transcription factor 5 (SOX5) and zinc finger and BTB domain containing 21 (ZBTB21) (Figure 2G). For the intronic lncRNAs, the expression patterns of 91 and only 2 were significantly positively and negatively correlated with their corresponding protein-coding genes, respectively (Figure S1A,B). For the SO lncRNAs, the expression patterns of 77 and only 2 were significantly positively and negatively correlated with their corresponding protein-coding genes, respectively (Figure S1C,D). Alluvial diagram analysis of our AS lncRNAs, intronic lncRNAs and SO lncRNAs showed that approximately 25% of these lncRNAs were significantly correlated (*p* < 0.05) with their corresponding protein-coding genes. AS lncRNA that were negatively correlated with their corresponding protein-coding genes were prone to enrich in the negative strand of the genome (Figure 2H). We allocated the lincRNAs to the flanking regions of annotated protein-coding genes and found that 2388 lincRNAs resided in the flanking regions of 7733 annotated protein-coding genes. The expression patterns of 731 lincRNA were significantly positively correlated with their flanking protein-coding genes and those of 92 lincRNA were significantly negatively correlated with their flanking protein-coding genes (Table S3). We identified that approximately 27% of total expressed lncRNAs were significantly correlated with their flanking (for lincRNAs) or reference (for intronic lncRNAs, SO lncRNAs and AS lncRNAs) genes.

### 3.3. Dynamics of lncRNA Expression during ARBW of Rabbit iATs

**Figure 3:**
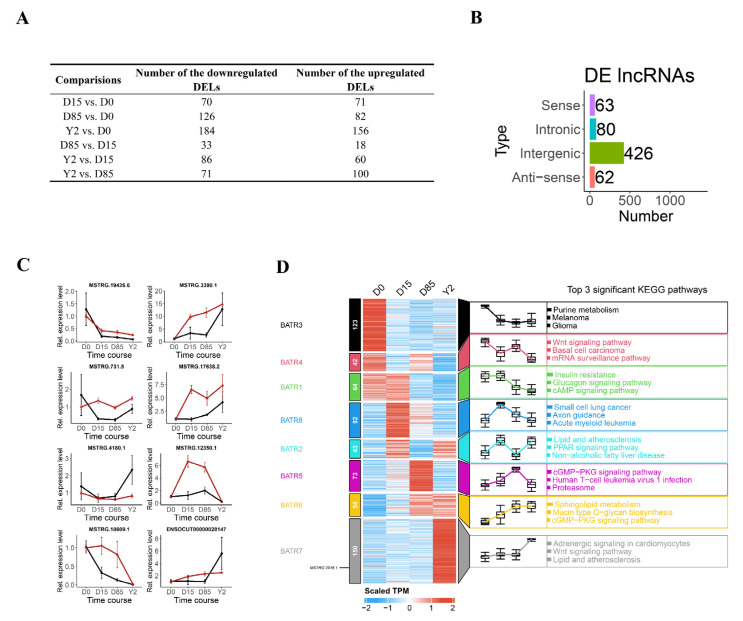
Dynamic changes and pathway enrichment of lncRNAs during the iAT whitening. (**A**) Counts of DELs in different comparisons. (**B**) The distribution of DELs in different lncRNA types. (**C**) Validation of DELs using qRT-PCR. The red line represents ssRNA-seq TPM values and the black line represents relative expression levels of qRT-PCR. The expression was normalized to the ACTB gene and D0. The data shows the means of three independent experiments. Two technical replicates were set for one individual experimental replicate. (**D**) Heatmap analysis of DELs based on k-means clustering method and KEGG pathway enrichment. The clusters are marked by different annotation blocks with different colors and the numbers of DELs in different clusters are showed in the left annotation blocks of the heatmap. The expression patterns of DELs in different clusters are shown in corresponding box-plots and top 3 significantly enriched KEGG pathways are shown.

When comparing iATs at different growth stages, a total of 70, 126, 184, 33, 86 and 71 lncRNAs were downregulated in the comparisons of D15 vs. D0, D85 vs. D0, Y2 vs. D0, D85 vs. D15, Y2 vs. D15 and Y2 vs. D85, respectively [log2(fold-change) > 1.5 and FDR < 0.01]. A total of 71, 82, 156, 18, 60 and 100 lncRNAs were upregulated in the comparisons of D15 vs. D0, D85 vs. D0, Y2 vs. D0, D85 vs. D15, Y2 vs. D15and Y2 vs. D85, respectively [log2(fold-change) < −1.5 and FDR < 0.01, Figure 3A]. Analysis of DELs found that the union set of 631 DELs in all comparisons contained 63 SO lncRNAs, 80 intronic lncRNAs, 426 lincRNAs and 62 AS lncRNAs (Figure 3B). To validate the ssRNA-seq results, we randomly selected eight DELs (MSTRG.19426.6, MSTRG.3390.1, MSTRG.731.5, MSTRG.17638.2, MSTRG.4180.1, MSTRG.12350.1, MSTRG.18609.1 and ENSOCUT00000028147) to evaluate their expression levels using qRT-PCR. The qRT-PCR results demonstrated that the expression patterns of all selected lncRNAs were consistent with those detected in the ssRNA-seq (Figure 3C and Table S4). Therefore, the results of reconstruction of transcriptome and quantification of lncRNA expression were reliable.

K-means clustering approach based on the TPM was used to sort all DELs, resulting in eight clusters, namely, BATR1 to BATR8, respectively (Figure 3D). The DELs in BATR3 and BATR4 were expressed in D0 and downregulated in D15. The KEGG pathway enrichment showed that purine metabolism and Wnt signaling pathway were the most significantly enriched pathways of DELs in BATR3 and BATR4, respectively. The DELs in BATR1, BATR2 and BATR8 were upregulated from D0 to D15 and downregulated in D85. KEGG pathway enrichment showed that DELs in BATR1, BATR2 and BATR8 were significantly enriched in the white adipose development-related pathways, such as insulin resistance, glucagon signaling pathway and peroxisome proliferator-activated receptor (PPAR) signaling pathway. The DELs in BATR5 and BATR6 were upregulated from D0 to D85. KEGG pathway enrichment showed that the cyclic guanosine monophosphate (cGMP)/cGMP-dependent protein kinase (cGMP-PKG) signaling pathway and sphingolipid metabolism were the most significantly enriched pathways of DELs in BATR5 and BATR6, respectively. cGMP-PKG was significantly enriched by the DELs in BATR6. The DELs in BATR7 were constantly expressed from D0 to D85 but dramatically upregulated in Y2. The KEGG pathway enrichment showed that the top3 significantly enriched pathways of DELs in BATR7 were adrenergic signaling in cardiomyocytes, Wnt signaling pathway and lipid and atherosclerosis (Figure 3D). The fatty acid metabolism and PPAR signaling pathway were also enriched by lncRNAs in BATR7.

### 3.4. Selection of lncRNA Candidates and Functional Validation of lncRNA MSTRG.2316.1

**Figure 4:**
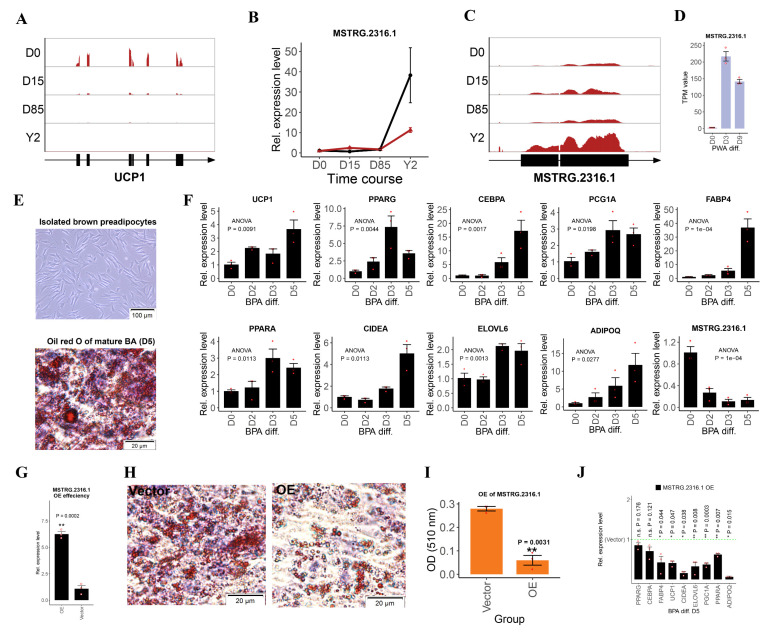
Selection of lncRNA candidates and functional validation of lncRNA MSTRG.2316.1. (**A**) The read coverage and gene structure of the UCP1 gene. The black blocks indicate the exons and the right direction of the arrow indicates the gene located in the positive strand. (**B**) qRT-PCR validation of lncRNA MSTRG.2316.1. The red line represents ssRNA-seq TPM values and the black line represents relative expression levels of qRT-PCR. (**C**) The read coverage and transcript structure of MSTRG.2316.1. (**D**) MSTRG.2316.1 upregulated during white adipocyte differentiation. (**E**) The upper panel indicates that the cell morphologies of the isolated rabbit brown preadipocytes are fusiform or triangular. The scale bar: 100 μm. The bottom panel indicates that the Oil red O staining identifies many lipid droplets in mature brown adipocytes after brown preadipocytes differentiating for 5 days. The scale bar: 20 μm. (**F**) Expression levels of BAT markers and MSTRG.2316.1 at 0 days (D0), 2 days (D2), 3 days (D3) and 5 days (D5) during rabbit brown adipocyte differentiation. One-way ANOVA analyses were performed and P values were marked in the figures. The “diff.” represented “differentiation”. The expression was normalized to the ACTB gene and D0. (**G**) Detection of OE efficiency after transfecting MSTRG.2316.1 plasmid vector. The expression was normalized to the ACTB gene and empty vector group. (**H**) Oil red O staining of lipid in cells that transfected MSTRG.2316.1 plasmid vector or empty plasmid vector at day 5. The left panel indicates that the cells are transfected with empty vector (empty vector group) and many lipid droplets have accumulated. The right panel indicates that cells are transfected with MSTRG.2316.1 plasmid vector (OE group) and brown adipogenesis is impaired. The scale bar: 20 μm. (**I**) Quantification of Oil red O at day 5. The data shows the means of three independent experiments and one technical replicate is set for one individual experimental replicate. (**J**) Determination of differences of gene expression levels of BAT markers between empty plasmid vector group and MSTRG.2316.1 plasmid vector group using qRT-PCR at day 5. The qRT-PCR data in (**B**,**F**,**G**,**J**) show the means of three independent experiments. Two technical replicates were set for one individual experimental replicate. The “*” represents *p* < 0.05 and “**” represents *p* < 0.01.

To focus on the efforts of lncRNA function validation, we ranked candidate lncRNAs by their abundance, differential expression during ARBW and significant correlation with their flanking or reference genes. A total of 10 lncRNA candidates were selected (Table S5). When searching the 10 lncRNAs to NONCODE database using BLAST, we found that 7 out of the 10 lncRNAs had significant sequence hits (Evalue < 1 × 10^−6^) in human and mouse lncRNAs, which might indicate the sequence conservation of lncRNAs between rabbits, mice and humans (Table S6). The histological analysis in this study (Figure 1A) and the BAT master marker UCP1 read coverage (Figure 4A) during the ARBW indicated that the BATs existed from D0 to D85. Only the samples in Y2 completed the whitening process, which spurred our interest in the cluster BATR7 and contained DELs expressed from D0 to D85, but dramatically upregulated in Y2 (Figure 3D). One lncRNA candidate, MSTRG.2316.1, in BATR7 with the highest TPM value in our lncRNA candidates was validated and its expression changed little from D0 to D85 and markedly upregulated 35 folds in Y2 in the qRT-PCR validation (Figure 4B). The 1449 bp length lincRNA MSTRG.2316.1 was located in chromosome 12, contained 2 exons on the positive strand of DNA and its read coverage dramatically increased in Y2 (Figure 4C). MSTRG.2316.1 was upregulated from D85 to Y2 during the ARBW of iATs and visceral white adipocyte differentiation, suggesting a potential negative regulator of brown adipocyte development (Figure 4D).

To validate the MSTRG.2316.1 functions, we first established the BPA differentiation model. Our results showed that BPAs isolated from iAT of rabbits in D0 were fusiform or triangular (Figure 4E upper panel). The Oil red O staining of the induced mature BATs showed that many lipid droplets had accumulated after differentiation for 5 days (Figure 4E bottom panel). The expression levels of common adipose markers, such as peroxisome proliferator activated receptor gamma (PPARG), CCAAT enhancer binding protein alpha (CEBPA), adiponectin C1Q and collagen domain containing (ADIPOQ) and fatty acid binding protein 4 (FABP4) and BAT-specific markers of cell death inducing DFFA like effector a (CIDEA), ELOVL fatty acid elongase 6 (ELOVL6), PPARG coactivator 1 alpha (PGC1A), peroxisome proliferator activated receptor alpha (PPARA) and UCP1 were significantly upregulated during differentiation of rabbit BPAs (Figure 4F). The expression levels of MSTRG.2316.1 were significantly downregulated after the induced differentiation of rabbit BPAs (Figure 4F).

To validate the function of MSTRG.2316.1, we performed gain-of-function analysis using plasmid vector-mediated overexpression (OE) of MSTRG.2316.1 in cultured BPAs. OE led to a significant 6 times increase of MSTRG.2316.1 level, compared to the cells treated with empty vector (Figure 4G). Oil red O results showed that OE of MSTRG.2316.1 impaired brown adipose differentiation, compared to transfection of empty plasmid vector (Figure 4H). Quantification of Oil red O of cells indicated that OE of MSTRG.2316.1 significantly decreased the lipid accumulation (*p* < 0.01 and Figure 4I). Additionally, the qRT-PCR analysis showed that OE of MSTRG.2316.1 significantly decreased FABP4, UCP1, CIDEA, ELOVL6, PCG1A, AIPOQ and PPARA expression (*p* < 0.05, Figure 4J). Our data showed that MSTRG.2316.1 was a negative regulator of BPA differentiation in vitro.

## 4. Discussion

The BAT histological characteristics had been depicted in some species, such as humans, mice and goats [6,11,40]. Our histological assays of rabbits suggested that the characteristics of rabbit BATs were in line with those of species showing the characteristics, including containing multiple small triglyceride droplets, the smaller size of cells and the highly expressed UCP1 and mitochondrial genes. BAT of most mammals undergoes ARBW. Previous studies have revealed the difference in the speed of ARBW among different species, such as hibernating animals persisting BAT into an adult, humans persisting race amounts of BAT into an adult and ruminants persisting BAT in 30 days after birth [6,10,40,41]. Our data revealed that the puberty rabbit contained a low proportion of multilocular adipocytes, suggesting that the ARBW of rabbits was slower than that of hibernating animals and ruminants and similar to that of humans. The long-time persisting BATs may explain the less fat-deposition in rabbits.

We identified 2281 credible novel lncRNAs by reconstructing the transcriptome, indicating the importance of lncRNAs in regulating the ARBW of rabbits. Intronic lncRNAs were transcribed from a single intron of protein-coding genes. Our data showed that intronic lncRNAs contained a few exons, which suggested that the exon number of lncRNAs might be affected by genomic elements of annotated protein-coding genes. Transcriptional complexity, expressed as the fraction of total RNAs, accounted for 10 or 100 most frequently expressed genes [42]. Our analysis revealed that classical BAT (iAT in D0) was a low lncRNA transcriptional complex tissue. On the other hand, the lncRNA transcriptional complexity between iATs and skeletal muscle tissues was similar and vWATs had a higher lncRNA transcriptional complexity than iATs and skeletal muscle tissues, which might suggest the transcriptomic difference between energy expenditure- and storage-related tissue.

Except for lincRNA, the AS lncRNA, intronic lncRNA and SO lncRNA were all transcribed from the loci of annotated protein-coding genes. Previous studies indicated that lncRNA transcribed from the loci of protein-coding genes might regulate the corresponding protein-coding genes [43,44]. For lincRNAs, we predicted their flanking protein-coding genes. By analyzing the expressional relationship between these three types of lncRNA and their corresponding protein-coding genes, we provided a valuable resource of the lncRNAs that were significantly correlated with their corresponding protein-coding genes, which can be used to identify functional lncRNAs efficiently.

In this study, analyses of lncRNA dynamics during rabbit iAT whitening were conducted. By comparing different stages during rabbit iAT development, we detected 631 DELs in the paired comparisons, which demonstrated that lncRNAs were widely involved in the BAT whitening in rabbits. We classified DELs into different clusters using the k-means method. The BAT is specialized for energy expenditure and heat generation, which depends on the function of mitochondria [45]. Purine nucleotides proved a constitutive inhibitor of UCP1 mediated proton conductance of the mitochondrial inner membrane and constituted the default shut-off mechanism in the absence of thermogenic demand [46]. Wnt signaling inhibits brown adipogenesis [47]. Our KEGG enrichment showed that the most significant pathway enriched by lncRNAs in BATR3 and BATR4 was purine metabolism and Wnt signaling pathway, which indicated that rapidly downregulated lncRNAs from D0 to D15 might mediate the ARBW through regulating their cis-regulated genes that were related to purine metabolism. A previous study indicated that a high-fat diet-induced BAT whitening and insulin resistance in mice [48]. In our study, the most significant KEGG pathway enriched by lncRNAs in BATR1, BATR2 and BATR8 were white adipose development-related pathways, such as insulin resistance and PPAR signaling pathway, suggesting the similarity pathway between obesity-related BAT whitening and ARBW and the lncRNA involvement. The cGMP-PKG pathway can promote the browning process of WATs. Our data indicated that the constantly upregulated lncRNAs from D0 to D85 were involved in this pathway, suggesting that these upregulated lncRNAs might play inhibitory roles during rabbit ARBW [49]. The KEGG enrichment showed that lncRNAs in BATR7 were widely involved in lipid metabolism pathways, such as lipid and atherosclerosis, fatty acid metabolism and PPAR signaling pathway, which indicated that this lncRNA might play crucial roles in white adipocyte development in the iATs of aged rabbits.

We characterized lncRNA MSTRG.2316.1 as a potential inhibitor that was upregulated in the iATs of aged rabbits and during differentiation of WATs. During the rabbit BPA differentiation, the common adipose markers and BAT-selective makers were all upregulated [50,51], which was similar to the previous mice BPA differentiation models. We established a robust rabbit BPA differentiation model. OE of MSTRG.2316.1 dramatically impaired the lipid accumulation in mature brown adipocytes and decreased the common adipose marker of FABP4 and the thermogenic marker genes (UCP1, CIDEA, ELOVL6, PGC1A and PPARA), indicating that MSTRG.2316.1 acted as an inhibitor during BAT development and might mediate the ARBW in aged rabbits. The molecular mechanisms underlying MSTRG.2316.1 regulating ARBW still need further investigation.

## 5. Conclusions

In summary, we provided comprehensive histological dynamics and detected a total of 2281 novel lncRNAs during ARBW of rabbit iATs. We revealed that classical rabbit BATs were low lncRNA transcriptional complex tissues. Dynamic analyses for lncRNAs identified 631 DELs during the ARBW. The purine metabolism pathways, Wnt signaling pathway, PPAR signaling pathway, cGMP-PKG signaling pathway and lipid and atherosclerosis were significantly enriched by the potential cis-regulated targets of the DELs with unique expression patterns during the ARBW. The novel identified lncRNA MSTRG.2316.1 can play an inhibitory role during brown adipocyte differentiation. Our work provides evidence that lncRNAs were widely involved in ARBW in rabbits, facilitating a better understanding of adipose biology.

## Data Availability

The datasets generated for this study can be found in the Sequence Read Archive (https://www.ncbi.nlm.nih.gov/sra, accessed on 10 November 2021) at NCBI, with the BioSample ID: SAMN18435507–SAMN18435518.

## Supplementary Materials

The following are available online at https://www.mdpi.com/article/10.3390/biology10111176/s1, Table S1: Primers of genes used in qRT-PCR (docx format); Table S2: Summary of ssRNA-seq data and transcriptome reconstruction (docx format); Table S3: LinRNAs that significantly correlated with their flanking protein-coding genes (xlsx format); Table S4: TPM values and differential analyses of DELs. Table S5. Information of 10 lncRNA candidates (docx format); Table S6: The NONCODE BLAST results of the 10 lncRNA candidates. Figure S1: Heatmap analyses of intronic lncRNAs and SO lncRNAs.

## References

[1] Peirce V., Carobbio S., Vidal-Puig A. (2014). The different shades of fat. Nature.

[2] Alvarez-Dominguez J.R., Bai Z., Xu D., Yuan B., Lo K.A., Yoon M.J., Lim Y.C., Knoll M., Slavov N., Chen S. (2015). De Novo Reconstruction of Adipose Tissue Transcriptomes Reveals Long Non-coding RNA Regulators of Brown Adipocyte Development. Cell Metab..

[3] van Marken Lichtenbelt W.D., Vanhommerig J.W., Smulders N.M., Drossaerts J.M., Kemerink G.J., Bouvy N.D., Schrauwen P., Teule G.J. (2009). Cold-activated brown adipose tissue in healthy men. N. Engl. J. Med..

[4] Saito M., Okamatsu-Ogura Y., Matsushita M., Watanabe K., Yoneshiro T., Nio-Kobayashi J., Iwanaga T., Miyagawa M., Kameya T., Nakada K. (2009). High incidence of metabolically active brown adipose tissue in healthy adult humans: Effects of cold exposure and adiposity. Diabetes.

[5] Bartelt A., Widenmaier S.B., Schlein C., Johann K., Goncalves R.L.S., Eguchi K., Fischer A.W., Parlakgül G., Snyder N.A., Nguyen T.B. (2018). Brown adipose tissue thermogenic adaptation requires Nrf1-mediated proteasomal activity. Nat. Med..

[6] Cypess A.M., Lehman S., Williams G., Tal I., Rodman D., Goldfine A.B., Kuo F.C., Palmer E.L., Tseng Y.H., Doria A. (2009). Identification and importance of brown adipose tissue in adult humans. N. Engl. J. Med..

[7] Derry D.M., Morrow E., Sadre N., Flattery K.V. (1972). Brown and white fat during the life of the rabbit. Dev. Biol..

[8] Lim S., Honek J., Xue Y., Seki T., Cao Z., Andersson P., Yang X., Hosaka K., Cao Y. (2012). Cold-induced activation of brown adipose tissue and adipose angiogenesis in mice. Nat. Protoc..

[9] Richard M.A., Pallubinsky H., Blondin D.P. (2020). Functional characterization of human brown adipose tissue metabolism. Biochem. J..

[10] Dawkins M.J., Hull D. (1964). Brown Adipose Tissue and the Response of New-Born Rabbits to Cold. J. Physiol..

[11] Schlein C., Fischer A.W., Sass F., Worthmann A., Tödter K., Jaeckstein M.Y., Behrens J., Lynes M.D., Kiebish M.A., Narain N.R. (2021). Endogenous Fatty Acid Synthesis Drives Brown Adipose Tissue Involution. Cell Rep..

[12] Batista P.J., Chang H.Y. (2013). Long noncoding RNAs: Cellular address codes in development and disease. Cell.

[13] St Laurent G., Wahlestedt C., Kapranov P. (2015). The Landscape of long noncoding RNA classification. Trends Genet..

[14] Kuang L., Lei M., Li C., Zhang X., Ren Y., Zheng J., Guo Z., Zhang C., Yang C., Mei X. (2018). Identification of Long Non-Coding RNAs Related to Skeletal Muscle Development in Two Rabbit Breeds with Different Growth Rate. Int. J. Mol. Sci..

[15] Robinson E.K., Covarrubias S., Carpenter S. (2020). The how and why of lncRNA function: An innate immune perspective. Biochim. Biophys. Acta Gene Regul. Mech..

[16] Peng W.X., Koirala P., Mo Y.Y. (2017). LncRNA-mediated regulation of cell signaling in cancer. Oncogene.

[17] Bai Z., Chai X.R., Yoon M.J., Kim H.J., Lo K.A., Zhang Z.C., Xu D., Siang D.T.C., Walet A.C.E., Xu S.H. (2017). Dynamic transcriptome changes during adipose tissue energy expenditure reveal critical roles for long noncoding RNA regulators. PLoS Biol..

[18] Mi L., Zhao X.Y., Li S., Yang G., Lin J.D. (2017). Conserved function of the long noncoding RNA Blnc1 in brown adipocyte differentiation. Mol. Metab..

[19] Ding C., Lim Y.C., Chia S.Y., Walet A.C.E., Xu S., Lo K.A., Zhao Y., Zhu D., Shan Z., Chen Q. (2018). De novo reconstruction of human adipose transcriptome reveals conserved lncRNAs as regulators of brown adipogenesis. Nat. Commun..

[20] Wang G.Z., Du K., Hu S.Q., Chen S.Y., Jia X.B., Cai M.C., Shi Y., Wang J., Lai S.J. (2018). Genome-wide identification and characterization of long non-coding RNAs during postnatal development of rabbit adipose tissue. Lipids Health Dis..

[21] Du K., Wang G.Z., Ren A.Y., Cai M.C., Luo G., Jia X.B., Hu S.Q., Wang J., Chen S.Y., Lai S.J. (2020). Genome-wide identification and characterization of long non-coding RNAs during differentiation of visceral preadipocytes in rabbit. Funct. Integr. Genom..

[22] Wang Z., He Z., Zhang D., Li H., Wang Z. (2020). Using oxidation kinetic models to predict the quality indices of rabbit meat under different storage temperatures. Meat Sci..

[23] Cullere M., Dalle Zotte A. (2018). Rabbit meat production and consumption: State of knowledge and future perspectives. Meat Sci..

[24] Dalle Zotte A., Szendro Z. (2011). The role of rabbit meat as functional food. Meat Sci..

[25] Wang G., Du K., Xie Z., Tang R., Jia X., Chen S., Lai S. (2020). Screening and Identification of Differentially Expressed and Adipose Growth-Related Protein-Coding Genes During the Deposition of Perirenal Adipose Tissue in Rabbits. Diabetes Metab. Syndr. Obes..

[26] Brown J., Pirrung M., McCue L.A. (2017). FQC Dashboard: Integrates FastQC results into a web-based, interactive, and extensible FASTQ quality control tool. Bioinformatics.

[27] Martin M. (2011). Cutadapt removes adapter sequences from high-throughput sequencing reads. Embnet J..

[28] Kim D., Langmead B., Salzberg S.L. (2015). HISAT: A fast spliced aligner with low memory requirements. Nat. Methods.

[29] Pertea M., Pertea G.M., Antonescu C.M., Chang T.C., Mendell J.T., Salzberg S.L. (2015). StringTie enables improved reconstruction of a transcriptome from RNA-seq reads. Nat. Biotechnol..

[30] Trapnell C., Roberts A., Goff L., Pertea G., Kim D., Kelley D.R., Pimentel H., Salzberg S.L., Rinn J.L., Pachter L. (2012). Differential gene and transcript expression analysis of RNA-seq experiments with TopHat and Cufflinks. Nat. Protoc..

[31] Kang Y.J., Yang D.C., Kong L., Hou M., Meng Y.Q., Wei L., Gao G. (2017). CPC2: A fast and accurate coding potential calculator based on sequence intrinsic features. Nucleic Acids Res..

[32] Wang L., Park H.J., Dasari S., Wang S., Kocher J.P., Li W. (2013). CPAT: Coding-Potential Assessment Tool using an alignment-free logistic regression model. Nucleic Acids Res..

[33] Sun L., Luo H., Bu D., Zhao G., Yu K., Zhang C., Liu Y., Chen R., Zhao Y. (2013). Utilizing sequence intrinsic composition to classify protein-coding and long non-coding transcripts. Nucleic Acids Res..

[34] Finn R.D., Bateman A., Clements J., Coggill P., Eberhardt R.Y., Eddy S.R., Heger A., Hetherington K., Holm L., Mistry J. (2014). Pfam: The protein families database. Nucleic Acids Res..

[35] Kutter C., Watt S., Stefflova K., Wilson M.D., Goncalves A., Ponting C.P., Odom D.T., Marques A.C., Bartel D.P. (2012). Rapid Turnover of Long Noncoding RNAs and the Evolution of Gene Expression. PLoS Genet..

[36] Yan P., Luo S., Lu J.Y., Shen X. (2017). Cis- and trans-acting lncRNAs in pluripotency and reprogramming. Curr. Opin. Genet. Dev..

[37] Love M.I., Huber W., Anders S. (2014). Moderated estimation of fold change and dispersion for RNA-seq data with DESeq2. Genome Biol..

[38] Gu Z., Eils R., Schlesner M. (2016). Complex heatmaps reveal patterns and correlations in multidimensional genomic data. Bioinformatics.

[39] Yu G., Wang L.G., Han Y., He Q.Y. (2012). clusterProfiler: An R package for comparing biological themes among gene clusters. Omics.

[40] Wang L., Yang X., Zhu Y., Zhan S., Chao Z., Zhong T., Guo J., Wang Y., Li L., Zhang H. (2019). Genome-Wide Identification and Characterization of Long Noncoding RNAs of Brown to White Adipose Tissue Transformation in Goats. Cells.

[41] Casteilla L., Champigny O., Bouillaud F., Robelin J., Ricquier D. (1989). Sequential changes in the expression of mitochondrial protein mRNA during the development of brown adipose tissue in bovine and ovine species. Sudden occurrence of uncoupling protein mRNA during embryogenesis and its disappearance after birth. Biochem. J..

[42] Mure L.S., Le H.D., Benegiamo G., Chang M.W., Rios L., Jillani N., Ngotho M., Kariuki T., Dkhissi-Benyahya O., Cooper H.M. (2018). Diurnal transcriptome atlas of a primate across major neural and peripheral tissues. Science.

[43] Cai R., Sun Y., Qimuge N., Wang G., Wang Y., Chu G., Yu T., Yang G., Pang W. (2018). Adiponectin AS lncRNA inhibits adipogenesis by transferring from nucleus to cytoplasm and attenuating Adiponectin mRNA translation. Biochim. Biophys. Acta Mol. Cell Biol. Lipids.

[44] You L., Zhou Y., Cui X., Wang X., Sun Y., Gao Y., Wang X., Wen J., Xie K., Tang R. (2018). GM13133 is a negative regulator in mouse white adipocytes differentiation and drives the characteristics of brown adipocytes. J. Cell. Physiol..

[45] Lai S.J., Du K., Shi Y., Li C., Wnag G.Z., Hu S.Q., Jia X.B., Wang J., Chen S.Y. (2020). Long Non-Coding RNAs in Brown Adipose Tissue. Diabetes Metab. Syndr. Obes..

[46] Bast-Habersbrunner A., Fromme T. (2020). Purine Nucleotides in the Regulation of Brown Adipose Tissue Activity. Front. Endocrinol. (Lausanne).

[47] Kang S., Bajnok L., Longo K.A., Petersen R.K., Hansen J.B., Kristiansen K., MacDougald O.A. (2005). Effects of Wnt signaling on brown adipocyte differentiation and metabolism mediated by PGC-1alpha. Mol. Cell. Biol..

[48] Kuipers E.N., Held N.M., In Het Panhuis W., Modder M., Ruppert P.M.M., Kersten S., Kooijman S., Guigas B., Houtkooper R.H., Rensen P.C.N. (2019). A single day of high-fat diet feeding induces lipid accumulation and insulin resistance in brown adipose tissue in mice. Am. J. Physiol. Endocrinol. Metab..

[49] Liu D., Ceddia R.P., Collins S. (2018). Cardiac natriuretic peptides promote adipose ‘browning’ through mTOR complex-1. Mol. Metab..

[50] Xiong Y., Yue F., Jia Z., Gao Y., Jin W., Hu K., Zhang Y., Zhu D., Yang G., Kuang S. (2018). A novel brown adipocyte-enriched long non-coding RNA that is required for brown adipocyte differentiation and sufficient to drive thermogenic gene program in white adipocytes. Biochim. Biophys. Acta Mol. Cell Biol. Lipids.

[51] da Silva I.V., Díaz-Sáez F., Zorzano A., Gumà A., Camps M., Soveral G. (2020). Aquaglyceroporins Are Differentially Expressed in Beige and White Adipocytes. Int. J. Mol. Sci..

