# Supplementary material for: De Novo Reconstruction of Transcriptome Identified Long Non-Coding RNA Regulator of Aging-Related Brown Adipose Tissue Whitening in Rabbits
Source: Biology (Basel). 2021 Nov 13;10(11):1176. doi: 10.3390/biology10111176 (PMC8614855; doi:10.3390/biology10111176)
Supplement: Supplementary file 1 [file biology-10-01176-s001.zip › Table S1.docx]

**Table S1 Primers of genes used in qRT-PCR**

| **Gene ID/siRNA** | **Ensembl ID** | **Primer (forward/reverse)** | **Product length (bp)** |
| --- | --- | --- | --- |
| *UCP1* | *ENSOCUG00000002297* | TGGGCACACTCGCATATTGT/TCGCTGGATTTTGCACAACG | 96 |
| *PPARG* | *ENSOCUG00000013194* | GAGGACATCCAGGACAACC/GTCCGTCTCCGTCTTCTTT | 168 |
| *PPARA* | *ENSOCUG00000009353* | ACAGGATTGATGCTGCCGAA/AAACGGATGTGCTGCCCTAA | 111 |
| *PGC1A* | *ENSOCUG00000014668* | AAAAGCTTGACTGGCGTCAC/ACTGCACCACTTGAGTCCAC | 202 |
| *CEBPA* | *ENSOCUG00000022985* | CAAGAACAGCAACGAGTACCG/GTCACTGGTCAACTCCAGCAC | 124 |
| *FABP4* | *ENSOCUG00000007445* | GGCCAGGAATTTGATGAAGTC/AGTTTATCGCCCTCCCGTT | 140 |
| *CIDEA* | *ENSOCUG00000011380* | TAGGGGACAACACGCACTTC/CTCTGGCGATTCCCGATCTC | 105 |
| *ELOVL6* | *ENSOCUG00000013069* | TCCTTTCTTCCACTTCCGGC/GAGTCGCTACGTGCTCTGTT | 133 |
| *CTTB* | *ENSOCUG00000029115* | ATGAATCGGAGGCCAACCAG/TTCGATTAGGCTTGCGAGGG | 112 |
| *COX2* | *ENSOCUG00000029099* | TCCGCATGCTAATCTCCTCG/TCCGGGAATGGCATCTGTTT | 83 |
| *ND1* | *ENSOCUG00000029086* | ACCCTAGCAGAAACCAACCG/TCCACATTGAAGCCGGAGAC | 77 |
| *ACTB* | *ENSOCUG00000005867* | GTGCTTCTAGGCGGACTGTT/CGGCCACATTGCAGAACTTT | 240 |
| *ADIPOQ* | *ENSOCUG00000026268* | TGTGCAGGTTGGATAGCAGG/ GAGCGGTAGACATAGGCACC | 233 |
| *MSTRG.19426.6* | *-* | TCGAGCATCCTATCCCCGAT/TGCACAGGGCTAATGTGAGG | 86 |
| *MSTRG.18609.1* | *-* | AGTCTCCGTTCTGGGGCTAA/CGGGAGAAGCATGCTCAGAA | 79 |
| *MSTRG.17638.2* | *-* | TGCTTCAGGCGTTCTGTGAT/GTGCCCCTCTTCTGCATTCT | 162 |
| *MSTRG.2316.1* | *-* | GACCGGACCCTTGGTCTATC/GCCTCTTCATTCTGGGGGAG | 104 |
| *MSTRG.3390.1* | *-* | CCGCTCATGGCAAACCATTT/AGATCCGCTCTCCAGAACCT | 135 |
| *MSTRG.731.5* | *-* | TCTGCTCATACCAAGGTGGA/ACAATGGAGATCATGCATCAGG | 92 |
| *MSTRG.4180.1* | *-* | GTGAACTGCCTGCCTACCTT/ATCCATTGCTCAGGGCACAA | 238 |
| *MSTRG.12350.1* | *-* | GGACGTCGGTAGGAGAAAGG/TAGCATCGCGTTTCCAAGGG | 199 |
| *ENSOCUT00000028147* | *ENSOCUT00000028147* | TCCCCCTTTTGTAGTTCAGTCC/ CCAGATGCAAGTCACACCCT | 195 |
