# Supplementary material for: De Novo Reconstruction of Transcriptome Identified Long Non-Coding RNA Regulator of Aging-Related Brown Adipose Tissue Whitening in Rabbits
Source: Biology (Basel). 2021 Nov 13;10(11):1176. doi: 10.3390/biology10111176 (PMC8614855; doi:10.3390/biology10111176)
Supplement: Supplementary file 1 [file biology-10-01176-s001.zip › Table S2.docx]

**Table S2** **Summary of ssRNA-seq data and transcriptome reconstruction**

| Sample | Clean reads | Clean reads Q20 (read1/read2) | Clean reads Q30 (read1/read2) | Total mapped reads | Uniquely mapped reads | Constructed transcripts number | Transcripts in forward strand |
| --- | --- | --- | --- | --- | --- | --- | --- |
| D0-1 | 113734636 | 98.82%/97.50% | 96.23%/92.96% | 108858150 (95.71%) | 89423364 (78.62%) | 59205 | 29408 |
| D0-2 | 127774702 | 98.85%/97.97% | 96.32%/94.11% | 122010174 (95.49%) | 97732248 (76.49%) | 58144 | 29001 |
| D0-3 | 144867602 | 98.83%/97.98% | 96.25%/94.12% | 138816469 (95.82%) | 110568612 (76.32%) | 58578 | 29065 |
| D15-1 | 108416600 | 98.86%/97.81% | 96.40%/93.80% | 101087388 (93.24%) | 84103748 (77.57%) | 58942 | 29370 |
| D15-2 | 112312308 | 98.78%/97.75% | 96.26%/93.82% | 105173940 (93.64%) | 89358348 (79.56%) | 67253 | 33597 |
| D15-3 | 116215322 | 98.85%/97.89% | 96.37%/94.05% | 109188631 (93.95%) | 90383528 (77.77%) | 66271 | 33160 |
| D85-1 | 121232316 | 98.86%/97.83% | 96.44%/93.86% | 113725153 (93.81%) | 94871142 (78.26%) | 63454 | 31713 |
| D85-2 | 137229838 | 98.84%/97.80% | 96.38%/93.50% | 129979890 (94.72%) | 114122186 (83.16%) | 66664 | 33352 |
| D85-3 | 119221610 | 98.82%/97.49% | 96.30%/93.81% | 111447944 (93.48%) | 93642294 (78.54%) | 63739 | 31951 |
| Y2-1 | 131058156 | 98.77%/97.49% | 96.26%/93.05% | 121970243 (93.07%) | 100894226 (76.98%) | 68909 | 34490 |
| Y2-2 | 122105490 | 98.78%/97.87% | 96.28%/94.02% | 113365466 (92.84%) | 92423324 (75.69%) | 67865 | 33874 |
| Y2-3 | 113739280 | 98.71%/97.53% | 96.06%/93.42% | 105033909 (92.35%) | 86291572 (75.87%) | 63800 | 31742 |
