# Supplementary material for: De Novo Reconstruction of Transcriptome Identified Long Non-Coding RNA Regulator of Aging-Related Brown Adipose Tissue Whitening in Rabbits
Source: Biology (Basel). 2021 Nov 13;10(11):1176. doi: 10.3390/biology10111176 (PMC8614855; doi:10.3390/biology10111176)
Supplement: Supplementary file 1 [file biology-10-01176-s001.zip › Table S5.docx]

| **LncRNA** | **Mean TPM** | **Chromosome** | **Exon number** | **Length** | **LncRNA type** | **Cluster** |
| --- | --- | --- | --- | --- | --- | --- |
| MSTRG.4180.1 | 9.66 | 13 | 5 | 1293 | lincRNA | 3 |
| MSTRG.2316.1 | 44.29 | 12 | 2 | 1449 | lincRNA | 7 |
| MSTRG.1263.1 | 9.98 | 1 | 3 | 764 | lincRNA | 5 |
| MSTRG.1331.2 | 10.08 | 10 | 2 | 4002 | lincRNA | 6 |
| MSTRG.18788.1 | 5.95 | GL019170 | 2 | 2445 | Intronic lncRNA | 7 |
| MSTRG.731.5 | 33.73 | 1 | 2 | 414 | Intronic lncRNA | 1 |
| MSTRG.11799.4 | 20.38 | 6 | 3 | 969 | SO lncRNA | 7 |
| MSTRG.16958.2 | 7.36 | GL018808 | 2 | 639 | SO lncRNA | 7 |
| MSTRG.18307.1 | 9.59 | GL019009 | 2 | 902 | SO lncRNA | 5 |
| MSTRG.4527.1 | 5.07 | 14 | 3 | 1160 | SO lncRNA | 8 |

**Table S4** **Information of 10 lncRNA candidates**
