# Supplementary figures and images for: De Novo Reconstruction of Transcriptome Identified Long Non-Coding RNA Regulator of Aging-Related Brown Adipose Tissue Whitening in Rabbits
Source: Biology (Basel). 2021 Nov 13;10(11):1176. doi: 10.3390/biology10111176 (PMC8614855; doi:10.3390/biology10111176)

A

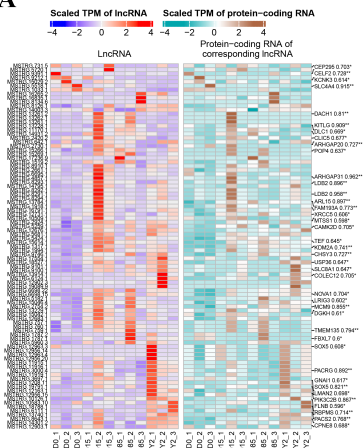

# B

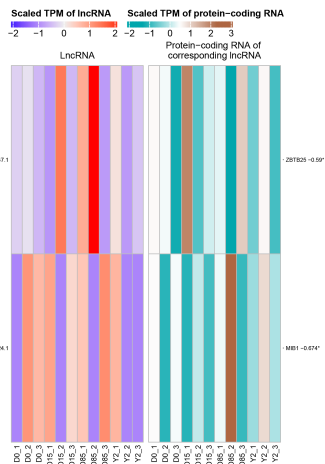

C

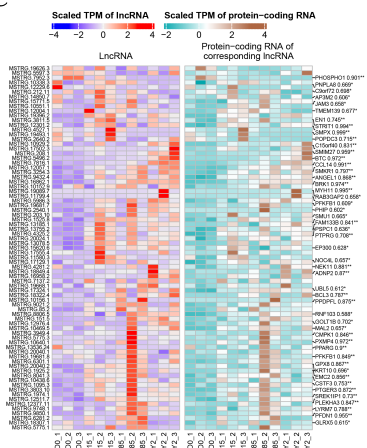

D

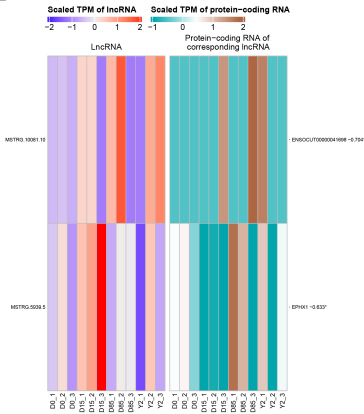

Supplement: Supplementary file 1 [file biology-10-01176-s001.zip › Figure S1.pdf]
